# Characterization of Dry Woven Fabrics Strengthened Polymer Concrete Composites

**DOI:** 10.1021/acsomega.5c02757

**Published:** 2025-05-22

**Authors:** Volkan Acar

**Affiliations:** Department of Mechanical Engineering, 37503 Atatürk University, Erzurum 25240, Türkiye

## Abstract

Polymer concretes (PCs) have recently attracted attention with their use in industry, thanks to their superior structural properties. To further improve the mechanical properties of polymer concretes, a kind of composite material, the achievements in composite technology should be transferred to this field. While studies in this context mainly involve dispersing chopped fibers in PC, the reinforcement of dry-woven fabric is not frequently encountered. In this study, the effects of the reinforcement of aramid, glass, and carbon dry woven fabrics under a specific layout plan on the flexural and compressive behaviors of polymer concrete were investigated. In this regard, first, the mechanical properties of the woven fabrics were analyzed by performing single yarn tensile, yarn pull-out, and woven tensile tests, and it was understood that aramid fabric has superior mechanical properties compared to other fabrics. After that, the reinforcement of PCs with dry woven fabrics was performed, and the density and mechanical tests were carried out parametrically at 7, 14, and 28 days of curing time. After 28 days of curing, a 14% increase in the flexural strength of carbon fabric-reinforced specimens occurred, while a 14% increase in the compressive strength of aramid fabric-reinforced specimens was obtained. The most important results of the study are that the reinforcement of polymer concrete with dry woven fabrics is quite effortless and manageable; the fibers carry the load and form a good interface with the polymer concrete, creating a bridging effect at the fracture and resulting in a strength increase.

## Graphical abstract

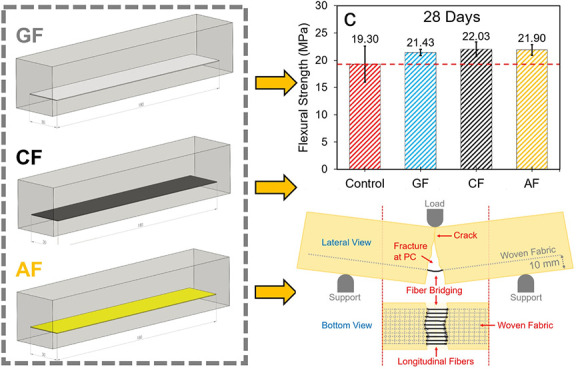

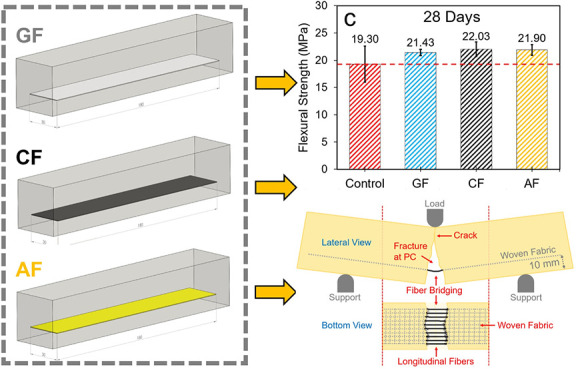

## 1 Introduction

Polymer concretes are composite materials consisting of aggregates, fillers, and polymerizing monomer binders.^1,2^ These products are frequently used in the construction and machinery industry. They are used in structural products such as underground pipes and slabs^3^ and as vibration-absorbing machine components.^4^ The mechanical properties of polymer concrete have been investigated by various parameters such as catalyst amount, resin type, and curing time.^5–7^

The mechanical properties can be enhanced through matrix modification in polymer composite research. In the studies conducted in this context, polymer concrete reinforced with chopped/short fibers is dominantly encountered. These fibers are generally carbon, glass, and basalt fibers. The fracture mechanics of polymer concrete reinforced with chopped/short fibers is the primary focus of these investigations. For example, an improvement of 3.4 times and 1.4 times in the fracture properties of specimens reinforced with chopped carbon fiber and chopped glass fiber, respectively, compared to the control specimen, was reported.^3^ In these studies, generally, favorable results were obtained with chopped/short fiber reinforcement, and fracture mechanics of polymer concretes were improved.^8–10^ On the other hand, it was noted that poor adhesion at the chopped fiber/polymer concrete interfaces causes fracture.^9^ In another study, chopped fibers restrained crack propagation by the fiber bridging mechanism and improved fracture toughness.^11^

The thermal management of chopped/short fiber-reinforced polymer concrete has also been investigated. The thermal stability of polymer concrete was increased by the reinforcement of chopped basalt fibers in a study.^12^ Significant improvements have been recorded in the fracture mechanics of chopped fiber-reinforced polymer concretes exposed to high temperatures.^11,13^

Another research topic in the field of fiber reinforcement for polymer concrete is the hybridization of chopped/short fibers. A study on the subject reported high flexural fatigue performance of hybrid chopped/short fiber-reinforced polymer concretes consisting of short steel and polypropylene fibers. In the same study, it was found that the curing time also affected the mechanical behavior of the polymer concrete. The strength of the reinforced polymer concretes was observed to be low in the early period compared to that of the unreinforced control specimens, while the strength was high after 28 days of curing. After curing, the flexural strength of the reinforced polymer concretes was calculated to be three times higher than that of the control specimens.^14^ In a study where similar chopped fibers were hybrid-reinforced, a similar increase in strength occurred after 28 days of curing.^15^

Chopped/short fiber-reinforced polymer concretes exposed to various environmental factors have also been the subject of research. In one of these studies, a 25% reduction in strength was observed in chopped glass fiber-reinforced polyester polymer concretes exposed to radiation for 8 weeks. In the same study, significant decreases in the strength of the mentioned polymer concrete exposed to aggressive environments such as acid and alkali were observed.^16^ Another study reported that the compressive strengths of chopped glass fiber-reinforced polymer concretes kept in sulfuric acid for 50 days remained at similar values to those of control specimens.^17^

Natural fibers have also been incorporated in chopped/short fiber-reinforced polymer concrete studies. In these studies, coconut, sugarcane bagasse, and banana fibers,^18^ wool and hemp fibers,^19^ and sisal and ramie fibers^20^ were utilized, and it was noted that natural chopped fiber reinforcement generally improved the mechanical properties of polymer concretes.

Up to this point, research on chopped/short fiber-reinforced polymer concrete composites, which are predominant in the existing reinforced PC literature, has been reviewed. On the other hand, it has been observed that woven fabrics have also been involved in polymer concrete studies in a very limited number. One of these studies investigated the compression behavior of tin slag polymer concretes externally wrapped with glass woven fabrics. It was reported that the compressive strength of the specimens increases as the number of wrapped layers increases, and the test speed affects the compressive strength.^21^ In a similar approach, in a study where carbon and glass woven fabrics were externally wrapped, it was reported that the carbon fiber reinforcement achieved a higher load-carrying capacity than glass fiber.^22^ Woven fabrics have also been employed in polymer concrete studies involving sandwich and panel structures. In these studies, polymer concrete was used as the core material, while woven jute fabrics were used as the face sheets, and the mechanical properties of the fabricated panels were investigated.^23,24^

It is understood from the literature review that chopped/short fiber and woven fabric reinforcement can significantly improve the mechanical properties of polymer concretes. In these studies, it was observed that chopped/short fibers were primarily utilized, while the use of woven fabrics was limited, and it was understood that polymer concretes could have superior mechanical properties with commercially available woven fabric reinforcement. In this research, the mechanical behavior of polymer concrete reinforced with aramid-, glass-, and carbon-woven fabrics was investigated. In this context, the fabrics cut to the specified dimensions were reinforced in polymer concrete specimens prepared in prism molds, and then, flexural, compression, and density tests were performed in accordance with the relevant standards. In addition, mechanical tests were carried out on the fabrics used in the study, and the mechanical properties underlying the behavior of these fabrics in polymer concrete were determined. As a result of these tests, the effects of the woven fabric reinforcement on the mechanical behavior of polymer concrete were analyzed comparatively.

## 2 Materials and Methods

### 2.1 Materials

The polymer resin was produced using polyester resin as the binder, quartz sand (specific gravity ∼ 2.65 g/cm^3^) as the aggregate, acetylacetone peroxide as the hardener, and cobalt as the accelerator. The aramid (AF), carbon (CF), and glass (GF) woven fabrics with a nominal weight of 200 g/m^2^ used as reinforcement materials in the study were supplied by Dost Kimya Inc. (Istanbul, Türkiye). Figure 1a shows the woven fabrics used in the study.

**Figure 1:**
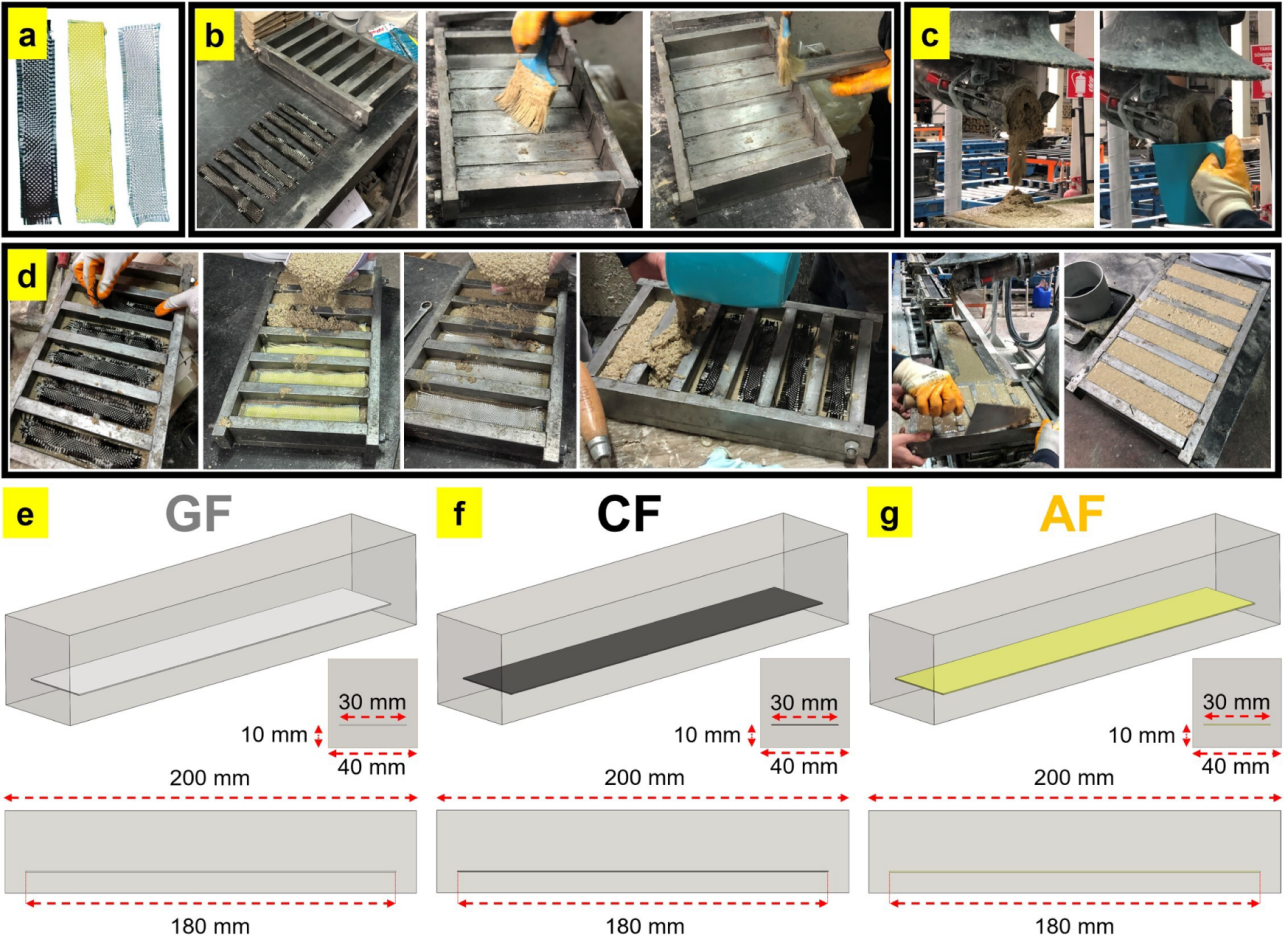
Production schematic of dry woven fabric reinforced polymer concretes: (a) dry woven fabrics used in the study, (b) preparation of molds for casting with mold release wax agent, (c) casting polymer concrete to a thickness of 10 mm above the bottom surfaces of the molds, (d) completion of molding by placing woven fabrics 10 mm above the bottom surface and casting polymer concrete back into molds; dimensions of the polymer concrete specimens: (e) GF, (f) CF, and (g) AF.

### 2.2 Preparation of Woven Fabric-Reinforced Polymer Concrete Specimens

The unreinforced polymer concrete production process, which was produced as a control specimen, was carried out using the process in ref 25 The polymer concrete used in the study consists of polyester resin (binder), acetylacetone peroxide (hardener), silica sand (0.3–3 mm, aggregate), and cobalt (accelerator). Molds with dimensions of 40 × 40 × 200 mm^3^ were used to produce woven fabric-reinforced polymer concrete prisms. In this context, woven fabrics were cut as 30 × 180 mm^2^ (Figure 1a). In the production of reinforced polymer concrete, a mold release wax agent was first applied to the mold surfaces to separate the cured polymer concrete from the steel mold easily. Polymer concrete was then cast up to 10 mm above the mold’s bottom surface. At this stage, the woven fabrics were placed individually, centered in width and length. Then, the molding process was completed by adding polymer concrete up to the top surface of the mold. The molding process was performed with the same spacing accuracy for all of the fabric types. After that, the products were subjected to initial curing in the molds for 15 min and then removed from the molds, and the curing process was continued under room conditions. In order to investigate the effect of curing time on the mechanical properties of polymer concrete, three curing times of 7, 14, and 28 days were determined, and the molds were left to cure under room conditions for the specified periods. Figure 1 shows the steps in the production process and the layout dimensions of the prisms and woven fabrics used in the study.

### 2.3 Single Yarn Tensile Test

Single yarn tensile tests were performed to determine the tensile performance of the yarns forming the fabrics used in the study. The gauge length was set at 150 mm in the tests. The yarn ends were glued with an epoxy-based adhesive between two soft textile fabrics to prevent the yarns from being pressed between the grip surfaces and creating a stress concentration (Figure 2a). Single yarn tensile tests were performed on a universal mechanical test machine (AG-IS Shimadzu Corp., Japan). Five specimens were tested at a 5 mm/min test speed and using a 5 kN load cell. Figure 2b shows single-thread tensile testing.

**Figure 2:**
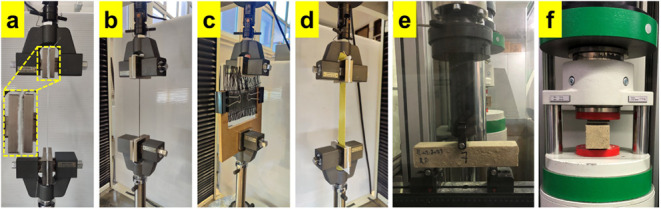
Mechanical tests on yarns/fabrics and polymer concrete specimens: (a) the soft textile fabric used to prevent specimens pressed during the tests, (b) the single yarn tensile test, (c) the yarn pull-out test, (d) the woven fabric tensile test, (e) three-point bending test, and (f) compression test.

### 2.4 Yarn Pull-Out Test

The yarn pull-out test was carried out using the universal test machine to determine the interyarn friction behavior of the fabrics used in the study. In this context, fabrics cut as 140 × 190 mm^2^ were placed between two wooden sheets, as seen in Figure 2c. The yarn pull-out tests, which are based on placing the wooden sheets in the lower grip and pulling a single yarn by the upper grip, were performed at a speed of 20 mm/min using a 5 kN load cell. At least five specimens were utilized in the yarn pull-out tests.

### 2.5 Woven Fabric Tensile Test

Woven fabric tensile tests were carried out using a universal test machine to determine the tensile properties of dry-woven fabrics. At least five specimens were used in the tests, and the gauge length was determined to be 150 mm, with a width of 30 mm. The test speed was 5 mm/min, and the load cell used was 5 kN. Fabric ends were glued with the adhesive between two soft textile fabrics, as was done in the single yarn tensile test. Figure 2d shows pictures of the tensile tests of woven fabrics.

### 2.6 Density, Flexural, and Compression Tests

Densities of the polymer concretes were measured in accordance with the TS EN 12390-7 ^26^ and ASTM C642 ^27^ standards by using three specimens for all PC composites. Flexural tests (three-point bending) of woven fabric-reinforced polymer concrete were performed according to TS EN 12390-5 ^28^ and ASTM C78/C78M,^29^ while compression tests were conducted according to TS EN 12390-3.^30^ The tests were carried out on a universal mechanical testing machine using a 600 kN load cell (a test speed of 0.05 kN/s), and three specimens were used for each test. The test procedure consists of two stages. In the first stage, flexural tests were carried out on fabric-reinforced polymer concretes. In the second stage, the prisms subjected to flexural tests were forced to separate from the center, and the remaining specimens were subjected to compression tests. Figure 2e,f shows the images of the flexural and compression tests.

## 3 Results and Discussion

### 3.1 Single Yarn Tensile Test Results

In single yarn tensile tests, the average maximum force values for GF, CF, and AF specimens were 81.27 ± 7.77, 212.31 ± 22.80, and 257.38 ± 13.12 N, respectively, and the average elongation at break values of these specimens were calculated as 2.68 ± 0.15%, 1.55 ± 0.05%, and 3.04 ± 0.28%, respectively. Figure 3a illustrates the test results. Aramid yarns showed remarkable tensile performance compared to that of other fabrics. This performance was followed relatively closely by carbon yarns. However, the maximum tensile strength of glass yarns was significantly lower than those of the other two fabrics. The average maximum tensile loads of aramid and carbon yarns were 216% and 161% higher than those of glass yarns, respectively. On the other hand, when the elongation at break under tensile load is considered, aramid yarns showed the best performance. Aramid yarns not only withstood higher tensile loads but also elongated more until rupture. It should be noted that carbon yarns showed elongation performance significantly lower than those of the other two fabrics despite reaching high tensile loads. Besides, glass yarns showed higher elongation compared to carbon yarns despite lower tensile loads. Figure 3b shows representative load–elongation curves of the yarns used in the tests. The GF, CF, and AF yarns were elongated by an average of 4.02 ± 0.23, 2.32 ± 0.08, and 4.56 ± 0.41 mm, respectively. Figure 4 shows the rupture of the yarns. As can be seen in the figure, the fibers in the cross sections of aramid and carbon yarns are not intensely ruptured, while deformations in the form of deterioration of these yarns and some local fiber ruptures are observed. On the contrary, the cross sections of the glass yarns showed extensive fiber ruptures, and the yarns often split in two.

**Figure 3:**
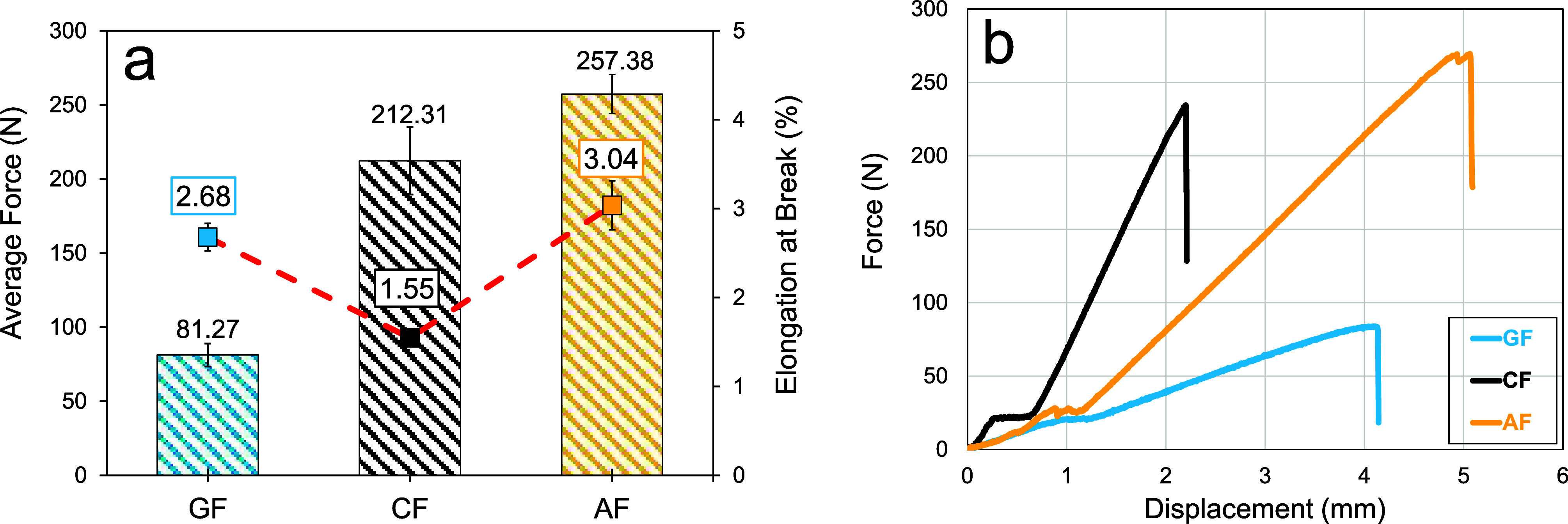
Single yarn tensile test results: (a) average forces and elongation at break values and (b) representative force vs displacement curves.

**Figure 4:**
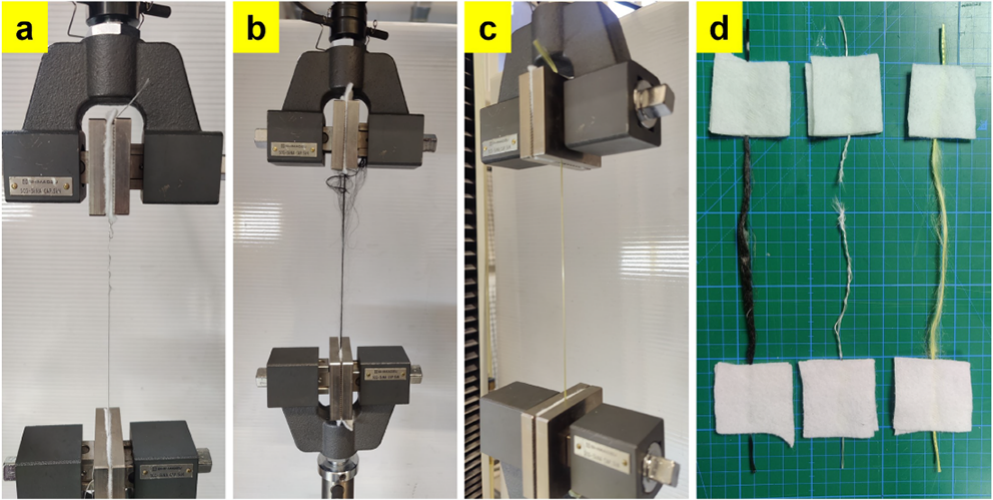
Images of deformations and ruptures in single yarn tensile tests: (a) GF yarn, (b) CF yarn, (c) AF yarn, and (d) ruptured yarns after the tests.

### 3.2 Yarn Pull-Out Results

Yarn pull-out testing helps to understand the interaction between the yarns that make up the fabrics. This interaction determines the mechanical properties of the fabric.^31^ The yarn friction performance of the fabrics was analyzed by comparing the average peak forces obtained from the test results. The peak force values, also defined as the maximum static frictional force,^32^ were determined to be 1.62 ± 0.24, 1.49 ± 0.20, and 2.28 ± 0.24 N for the GF, CF, and AF specimens, respectively (Figure 5a). The yarn pull-out performance of the aramid fabric is remarkable compared to other fabrics. It was found that the yarn pull-out load of aramid fabrics was, on average, 40% and 53% higher than those of glass and carbon fabrics. These test results do not show a trend similar to that of the single yarn tensile test results. While the tensile load of carbon yarn is considerably higher than that of glass yarn, the static friction force of carbon fabric is slightly lower than that of glass fabric. This resulted in the high-performance difference between carbon and glass in the single yarn tensile tests being reduced in the woven tensile tests. Figure 5b–d shows the representative pull-out force–displacement curves of the yarns, while Figure 6 shows the specimens subjected to the yarn pull-out test.

**Figure 5:**
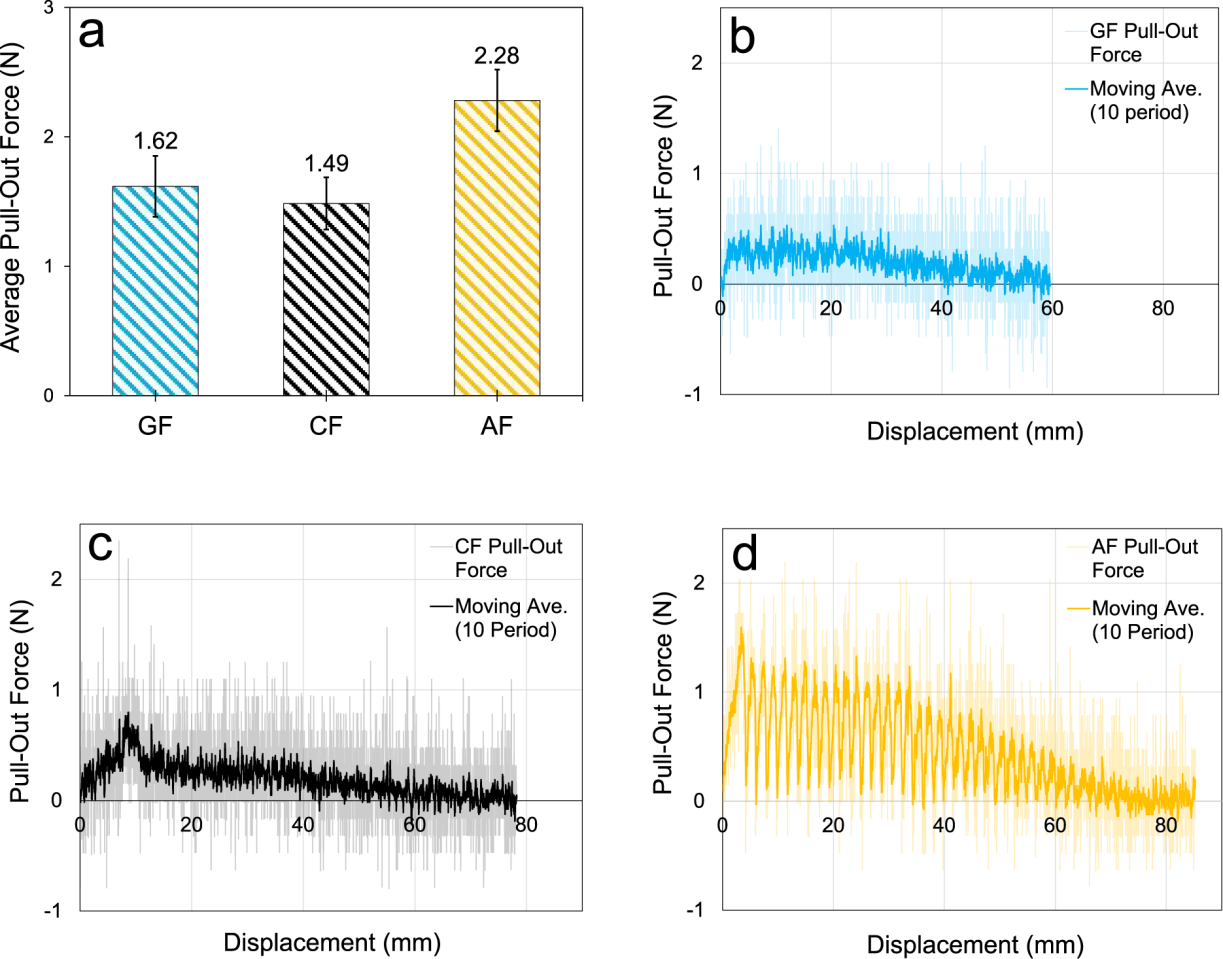
Yarn pull-out test results: (a) average pull-out forces, (b) representative force vs displacement of GF, (c) representative force vs displacement of CF, and (d) representative force vs displacement of AF.

**Figure 6:**
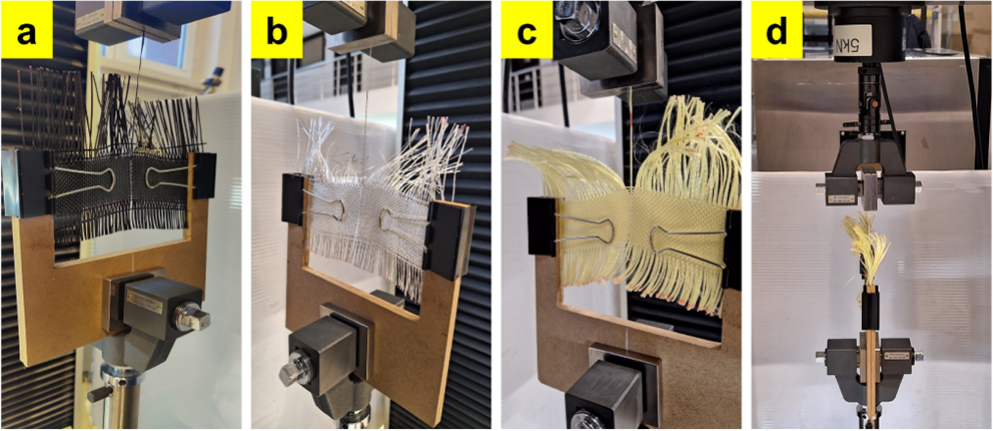
Images of the deteriorations in the yarn pull-out tests: (a) CF, (b) GF, (c) AF, and (d) a transverse test image showing pressed wooden sheets.

### 3.3 Woven Fabric Tensile Test Results

In woven fabric tensile tests, average maximum tensile loads of 1013.03 ± 127.56, 1351.13 ± 75.85, and 1621.84 ± 198.67 N were obtained for GF, CF, and AF fabrics, respectively. The elongation at break of these fabrics was calculated to be 3.47 ± 0.29%, 2.36 ± 0.11%, and 5.97 ± 1.09%, respectively. The relevant results are listed in Figure 7a. Aramid fabric showed superior performance in both woven fabric tensile and single yarn tests. These results show that fabrics consisting of yarns with a higher load-carrying capacity show better tensile behavior. Accordingly, aramid and carbon fabrics carried 60% and 33% more load than glass fabrics, respectively. The performance differences in woven fabric tensile tests were reduced compared to single yarn tensile tests. This can be explained by the relatively adequate performance of the glass fabric in yarn pull-out tests. The elongation at break results of the fabrics is similar to the trend in single yarn tensile tests. Accordingly, aramid fabrics were the most elongated specimens until rupture, while carbon fabrics showed low elongation. Glass fabrics showed higher elongation than their carbon counterparts. Figure 7b shows representative load–displacement curves obtained from woven fabric tensile tests. Aramid fabrics have attracted attention with their high load-carrying capacity and displacement values. Figure 8 shows the rupture of the woven fabrics. In carbon fabrics, severe distortions and separations between the yarns were observed during shrinkage, and it was found that these deformations disrupted the weave structure of the fabric. This behavior is consistent with the low yarn pull-out test results of carbon fabrics. Similar behavior, but to a lesser extent, is also observed in glass fabrics. However, the main problem with glass fabrics is that the glass yarns rupture considerably, and the fabric can no longer be load bearing. The rupture behavior of aramid fabrics is demonstrated by the rupture of local yarns and the same limited local zones of the fabric cross-section becoming unable to bear the load.

**Figure 7:**
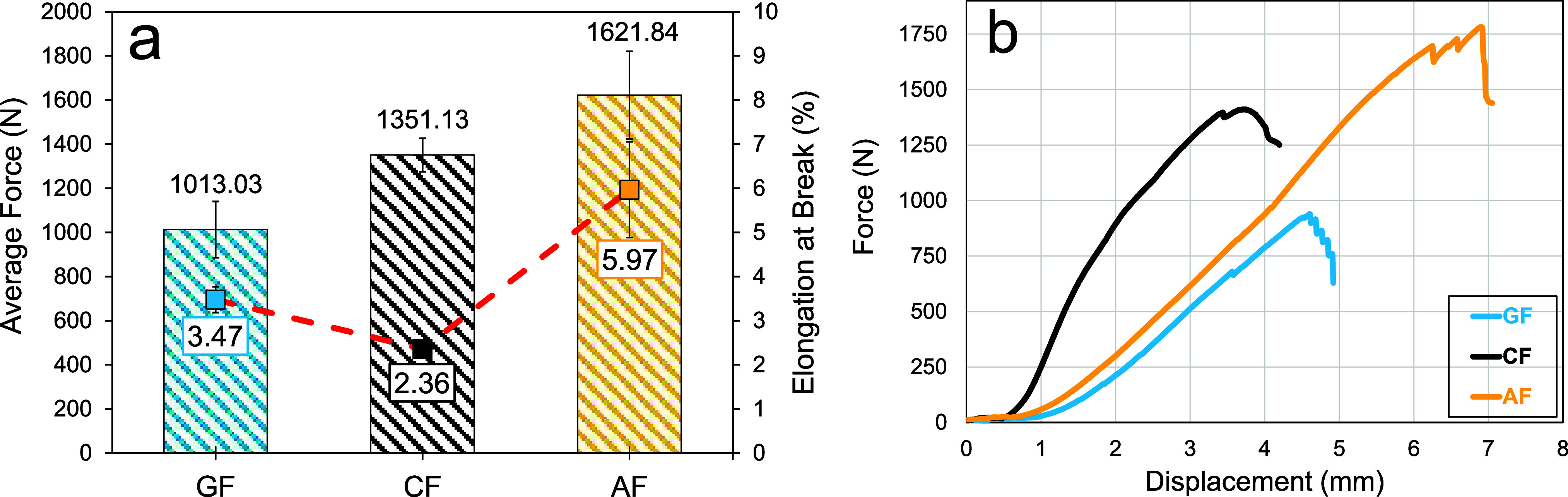
Woven fabric tensile test results: (a) average forces and elongation at break values and (b) representative force–displacement curves.

**Figure 8:**
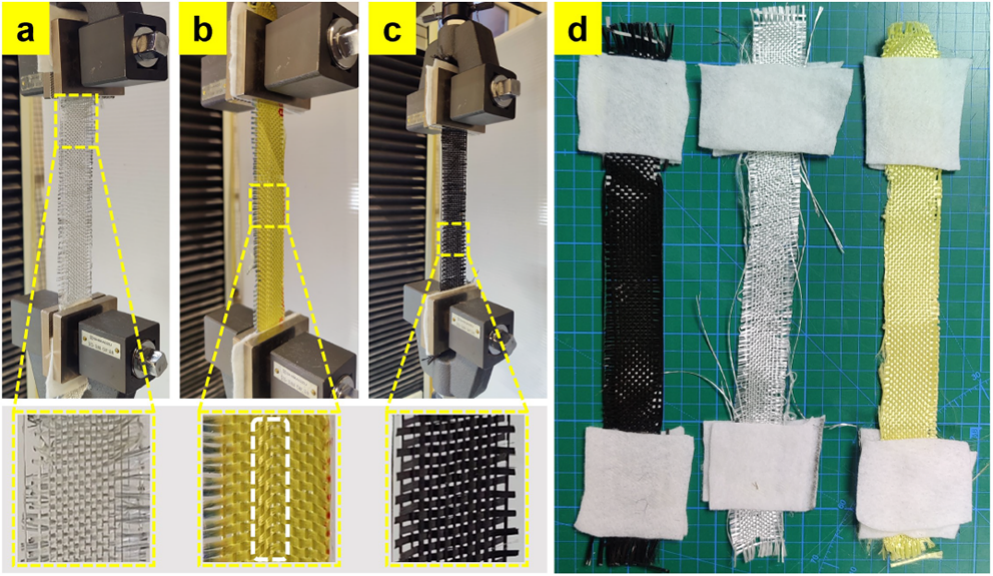
Images of the deteriorations in the woven fabric tensile tests: (a) GF, (b) AF, (c) CF, and (d) top view of ruptured fabrics.

### 3.4 Density Measurements

Figure 9 shows the average density results and graphs of the measured densities depending on the curing time. The density measurements showed similar values for the densities of all PC groups. After 28 days of curing, it was determined that the sample densities were in the range ∼2118–2184 kg/m^3^ on average. On the other hand, it was also understood that the densities of carbon fabric-reinforced specimens were slightly higher compared to other fabrics at all curing times. The increase in density of the carbon fabric-reinforced PCs can be explained by the fact that the void fraction in these specimens is lower, and therefore, the carbon fabric has better adhesion with the polymer resin. According to these results, after 28 days of curing, the average densities of the carbon fabric-reinforced specimens were 1.26%, 3.13%, and 1.79% higher than those of the control, GF, and AF specimens, respectively. After 28 days of curing, the lowest average density value was observed in the GF specimens. The average density values of the GF specimens were calculated to be 1.82%, 3.04%, and 1.30% less than the control, CF, and AF specimens, respectively. It is thought that there is poor adhesion at the interface of glass fabric and polymer concrete compared to other fabrics and that there are more voids at the interface. The density data of the specimens are given in Tables 1–3.

**Figure 9:**
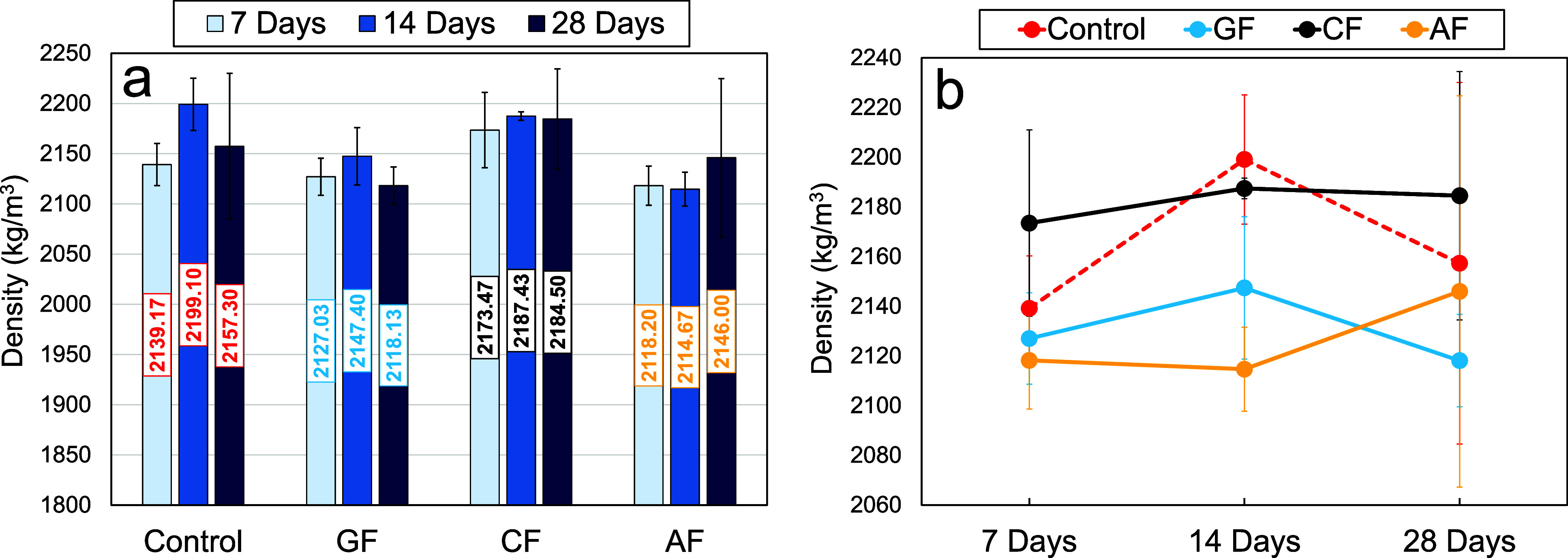
(a) Average density values of the PCs and (b) variation of density values depending on the curing times.

**Table 1:** Test Results After 7 Days of Curing

Specimen	No	Density [kg/m^3^]	Flexural Strength [MPa]	Compression Strength 1 [MPa]	Compression Strength 2 [MPa]
Control	1	2141.80	19.40	89.30	89.30
	2	2116.90	19.70	89.60	91.00
	3	2158.80	19.80	87.40	88.70
	Ave.	2139.17 ± 21.07	19.63 ± 0.21	89.22 ± 1.18	
GF	1	2106.30	19.30	91.70	88.80
	2	2141.30	18.70	85.00	87.30
	3	2133.50	18.30	83.70	86.30
	Ave.	2127.03 ± 18.37	18.77 ± 0.50	87.13 ± 2.85	
CF	1	2149.90	21.80	94.30	92.50
	2	2153.80	21.30	90.00	90.40
	3	2216.70	21.10	92.60	93.10
	Ave.	2173.47 ± 37.49	21.40 ± 0.36	92.15 ± 1.65	
AF	1	2134.90	18.80	87.20	86.40
	2	2096.70	17.80	82.10	85.50
	3	2123.00	19.40	82.80	86.60
	Ave.	2118.20 ± 19.55	18.67 ± 0.81	85.10± 2.14	

**2:** Test Results After 14 Days of Curing

Specimen	No	Density [kg/m^3^]	Flexural Strength [MPa]	Compression Strength 1 [MPa]	Compression Strength 2 [MPa]
Control	1	2229.10	21.20	81.60	82.70
	2	2185.60	22.10	75.00	85.20
	3	2182.60	19.40	93.00	87.50
	Ave.	2199.10 ± 26.02	20.90 ± 1.37	84.17 ± 6.05	
GF	1	2168.10	21.40	87.30	87.30
	2	2114.70	18.90	90.70	89.90
	3	2159.40	19.60	90.30	88.80
	Ave.	2147.40 ± 28.65	19.97 ± 1.29	89.05 ± 1.50	
CF	1	2184.60	21.50	86.90	89.10
	2	2185.50	21.00	86.30	88.60
	3	2192.20	21.60	97.90	89.20
	Ave.	2187.43 ± 4.15	21.37 ± 0.32	89.67 ± 4.21	
AF	1	2118.40	19.50	95.60	94.90
	2	2096.20	19.30	94.00	93.90
	3	2129.40	20.30	91.10	91.10
	Ave.	2114.67 ± 16.91	19.70 ± 0.53	93.43 ± 1.91	

**Table 3:** Test Results After 28 Days of Curing

Specimen	No	Density [kg/m^3^]	Flexural Strength [MPa]	Compression Strength 1 [MPa]	Compression Strength 2 [MPa]
Control	1	2073.30	19.50	82.20	85.80
	2	2200.60	15.90	82.90	84.30
	3	2198.00	22.50	85.90	86.70
	Ave.	2157.30 ± 72.76	19.30 ± 3.30	84.63 ± 1.80	
GF	1	2139.50	21.60	93.90	95.30
	2	2105.40	20.80	93.60	95.20
	3	2109.50	21.90	92.00	96.40
	Ave.	2118.13 ± 18.62	21.43 ± 0.57	94.40 ± 1.56	
CF	1	2198.60	22.00	92.30	93.30
	2	2225.90	23.40	98.30	92.30
	3	2129.00	20.70	89.60	93.80
	Ave.	2184.50 ± 49.97	22.03 ± 1.35	93.27 ± 2.86	
AF	1	2160.20	21.60	95.20	98.40
	2	2061.10	21.10	97.50	98.10
	3	2216.70	23.00	93.40	97.60
	Ave.	2146.00 ± 78.77	21.90 ± 0.98	96.70 ± 1.97	

### 3.5 Flexural Test Results

The average flexural strengths of the control, AF, GF, and CF specimens were determined as 19.63 ± 0.21, 18.67 ± 0.81, 18.77 ± 0.50, and 21.40 ± 0.36 MPa, respectively, at the end of the 7 day curing period. A trend similar to the 7 day density results of the specimens was seen in the flexural strengths obtained after 7 days of curing. It was found that the average flexural strength of the CF specimens was 9% higher than that of the control specimens. After 14 days of curing, the average flexural strengths of the specimens were 20.90 ± 1.37, 19.70 ± 0.53, 19.97 ± 1.29, and 21.37 ± 0.32 MPa for the control, AF, GF, and CF specimens, respectively. At this point, it is understood that the difference in the flexural strength between the CF and control specimen groups decreased. However, the trend of poor flexural performance in the AF and GF specimen groups continues. A similar continuity between the trend of density values and flexural strength is observed at the end of 14 days. After 28 days of curing, average flexural strengths were 19.30 ± 3.30, 21.90 ± 0.98, 21.43 ± 0.57, and 22.03 ± 1.35 MPa for the control, AF, GF, and CF specimens, respectively. According to these results, the average flexural strength of the fabric-reinforced PCs increased. Accordingly, the average flexural strengths of the CF, AF, and GF specimens were found to be approximately 14%, 13%, and 11% higher than those of the control specimens, respectively. On the other hand, the high standard deviation of the average flexural strength result of the control specimen is also noteworthy. It is considered that there are two reasons for the high flexural performance of the CF specimens. First, the high tensile strength of carbon fabric and second, as seen in the density results, show the best adhesion performance and form a better interface with polymer concrete. A similar study^33^ reported that the flexural behavior of specimens with basalt mesh placed in different configurations between polymer concrete layers was improved compared to unreinforced specimens. In the same study, the same reinforcement process was also applied to ordinary cement-based concretes, and it was observed that polymer concretes with and without basalt mesh reinforcement exhibited superior flexural performance. This superior performance is more noticeable in reinforced polymer concretes. The significant improvement is because of the enhanced adhesion of basalt fibers to the polymer compared to that of ordinary cement. With a curing time of 7 days, the flexural strength of the polymer concrete control specimen remained almost constant at the end of 28 days, while a slight increase was observed in the fabric-reinforced specimens. In the literature, it was reported that the mechanical properties of polymer mortars reach a certain value and remain almost constant with a curing time of 7 days, while this period is longer for conventional cement mortars.^34^ This time advantage is one of the parameters in which polymer concrete is superior to ordinary cement-based concretes. In this study, the flexural strengths of all different types of specimens reached a certain value within 7 days of curing time and changed slightly with increasing curing time. Accordingly, after 7 days of curing, it can be seen that a good adhesion and interface have not yet formed between the polymer concrete and the fabrics. Nevertheless, it should be noted that, as already mentioned, the CF fabric forms a better adhesion and interface with the PC compared to other fabrics with a 7-day cure. After 14 days of curing, the degree of adhesion between the fabrics and the polymer concrete gradually increased, and after 28 days of curing, it was observed that the interfaces were stabilized, and the flexural strength increased due to the strong adhesion formed. The high mechanical properties of aramid and carbon fabrics in the mechanical tests of fabrics used as reinforcements in PCs have been discussed previously. At the end of 28 days of curing, specimens reinforced with these fabrics showed slightly higher strength performance than glass fabric specimens in flexural tests, which can be attributed to this reason.

The higher yarn pull-out performance of glass fabric compared to carbon fabric may explain the increase in flexural strength of glass fabric specimens due to the higher friction between the yarns than that of carbon yarns. On the other hand, it can be said that the aramid fabric, with its high tensile strength and maximum yarn pull-out load, endures normal stresses due to bending considerably well. The fact that carbon fabric provides higher flexural strength with less fabric tensile strength compared to that of aramid fabric demonstrated that carbon fabric has better adhesion with the polymer concrete matrix. The increase in density of the CF-reinforced specimens also indicates that, as previously mentioned, there are fewer voids at the interface between these fabrics and the polymer concrete compared with other fabrics and that this adhesion is more effective than with other fabrics.

The increase in flexural performance is considered to be due to two reasons: (i) the load-bearing capacity of the woven fabrics and (ii) the bridging effect of the woven fabrics. First, the woven fabrics carry the load during bending, resulting in less normal stress in the cross-section of the reinforced specimens than in the cross-section of the control specimen. Second, the bridging effect occurs since the fabrics do not rupture during crack initiation and propagation in the PC, and fabrics still carry the load between the two fractured parts of the polymer concrete (Figure 10). As seen in Figure 10, after the fracture in the polymer concrete, the longitudinal fibers of the woven fabrics are not ruptured, and a bridging mechanism occurs between the two fractured parts. This mechanism continues to carry the load for a certain period. For these two reasons, the load-bearing capacity and flexural strength of polymer concrete increase. In a study^35^ in which cementitious concretes were reinforced with single-layer carbon fiber/epoxy prepregs in different configurations internally and externally, it was reported that reinforcement from the midplane of the prism increased the flexural strength of the concrete by 30.21%. The reason the increase in flexural strength in the mentioned study is higher than in this study is that the epoxy-impregnated carbon fiber prepregs have a higher tensile strength compared to dry fabrics.

**Figure 10:**
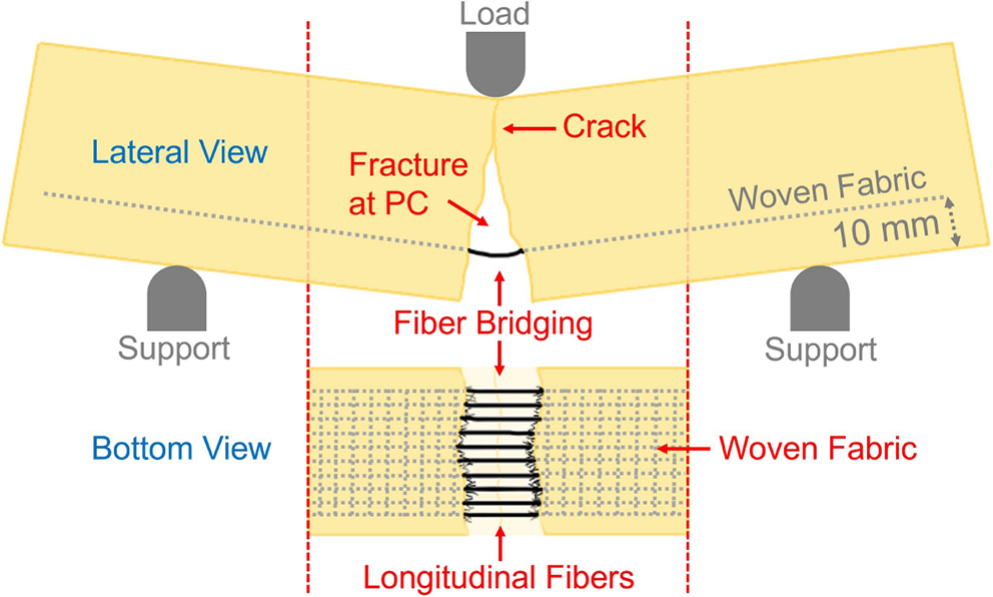
Fiber bridging mechanism in an unsplit flexural specimen.

One of the important parameters in strengthening processes with fiber-reinforced composite (FRP) is the fiber/concrete interface.^36,37^ The degree and strength of interfacial bonding directly affect the strengthening efficiency and also directly affect the configuration of reinforcement scenarios. In this context, the study^35^ also reported that internal reinforcements showed better flexural performance than external reinforcements. It was also reported that the composite debonded more easily from concrete in external reinforcements, and sliding was observed at the interface of prepreg and concrete in internal reinforcements due to the low thickness of the prepregs. In this study, such a situation was not observed when the fractured interfaces were analyzed. This is because the woven fabrics used in the study were much thicker than the prepregs (0.125 mm), and the dry-woven fabrics formed a better bond with the polymer concrete compared to the prepregs with cured epoxy surfaces. However, as mentioned before, the high tensile strength of the prepregs increased the flexural strength of the concrete more as a result. In a study on the flexural creep behavior of fiber-reinforced concrete, it was reported that two layers of glass fabric/epoxy composite reinforcement prevented early fracture of concrete.^38^ In another study, mechanical analyses of cements reinforced with polyester and flax woven fabrics were performed, and it was stated that polyester fabrics showed better performance than flax fabrics.^39^ The average flexural strengths of PCs depending on the curing times are presented in Figure 11. All of the data of the flexural tests are given in Tables 1–3.

**Figure 11:**
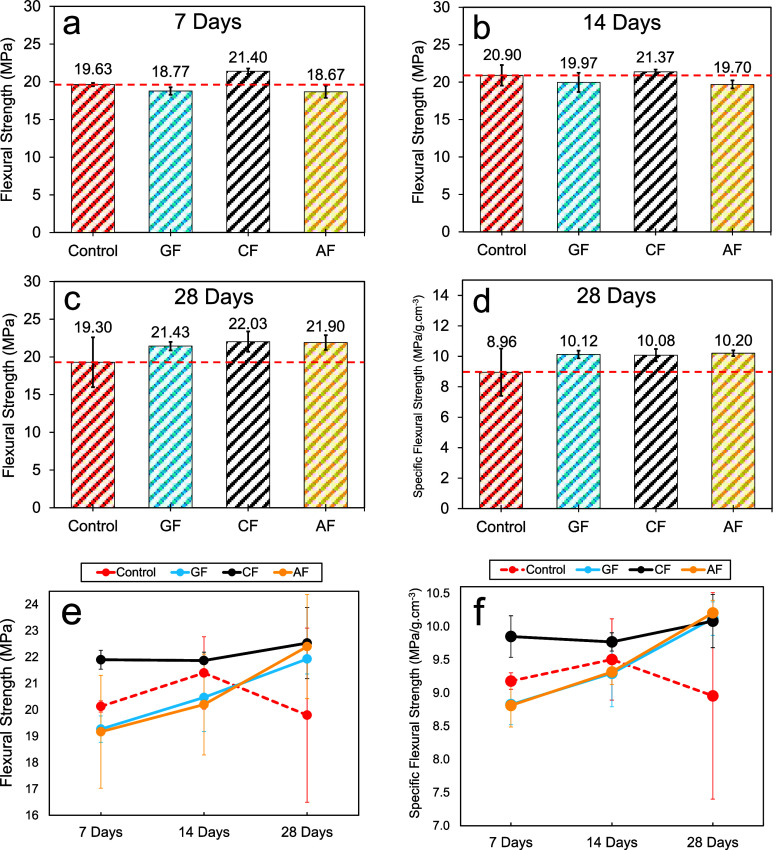
Average flexural strengths: (a) 7 days, (b) 14 days, (c) 28 days, (d) specific flexural strength for 28 days, (e) flexural strength variations depending on curing times, and (f) specific flexural strength variations depending on curing times.

Figure 12 shows images of a representative fractured specimen after the flexural test. Figure 12a shows that the crack initiates from the bottom surface of the prism and that the specimen is not completely fractured. The fiber bridging effect can be seen in Figure 12b,c. It is understood that the fabrics work together with the polymer concrete, preventing the specimen from splitting into two. In these images, it can also be noted that the fabrics are not ruptured but rather elongated. A previous study reported that in flexural tests of cement reinforced with recycled poly­(ethylene terephthalate) fabrics, the specimens showed no separation at the end of the test and remained together.^40^ Figure 12d,e shows the cross-sectional image of the specimen, which was split in two, after it was forced to fracture by hand. In this image, it is seen that the fabrics have good adhesion with the polymer concrete. The fracture behavior in the selected image was similar for all three fabric types.

**Figure 12:**
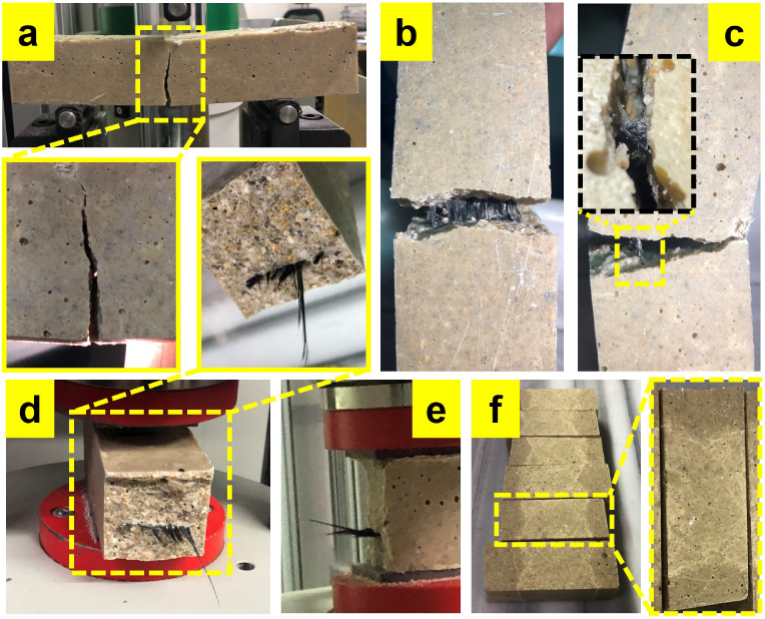
Fracture images after the flexural (FT) and compression (CT) tests: (a) crack propagation in FT, (b) bridging effect in FT, (c) close-up view of the bridging effect in FT, (d) fabric/PC interface at the CT test specimen and cross-sectional view of the specimen fractured by hand after the FT, (e) CT specimen during the test, and (f) surfaces subjected to the compression.

### 3.6 Compression Test Results

Tables 1–3 provide compression test data. Figure 13 shows average compressive strength graphs based on three different curing times. According to these results, the average compressive strengths of the control, AF, GF, and CF specimens at the end of the 7-day curing time were determined to be 89.22 ± 1.18, 85.10 ± 2.14, 87.13 ± 2.85, and 92.15 ± 1.65 MPa, respectively. These results show a trend similar to that of the 7 day density values. In the test results after this curing time, the average compressive strength of the CF specimens is 3% higher than that of the control specimen, as are the flexural strength values. The 14 day test results were 84.17 ± 6.05, 93.43 ± 1.91, 89.05 ± 1.50, and 89.67 ± 4.21 MPa for the control, AF, GF, and CF samples, respectively. After 14 days of curing, the compressive strength of the fabric-reinforced specimens increased. Remarkably, the average compressive strength of the AF specimens was 11% higher than that of the control specimen. On the other hand, the AF specimens exhibited an opposite behavior between the low-density value obtained after 14 days of curing and the compressive strength performance. This is thought to be due to the fact that the compaction during the compression test reduces the number of voids caused by the relatively weak adhesion. After 28 days of curing, the positive performance trend of the AF specimens continued in the compression tests. The 28 day test results were 84.63 ± 1.80, 96.70 ± 1.97, 94.40 ± 1.56, and 93.27 ± 2.86 MPa for the control, AF, GF, and CF specimens, respectively. From these results, the increase was calculated to be 14%, 11%, and 10% for AF, GF, and CF specimens, respectively.

**Figure 13:**
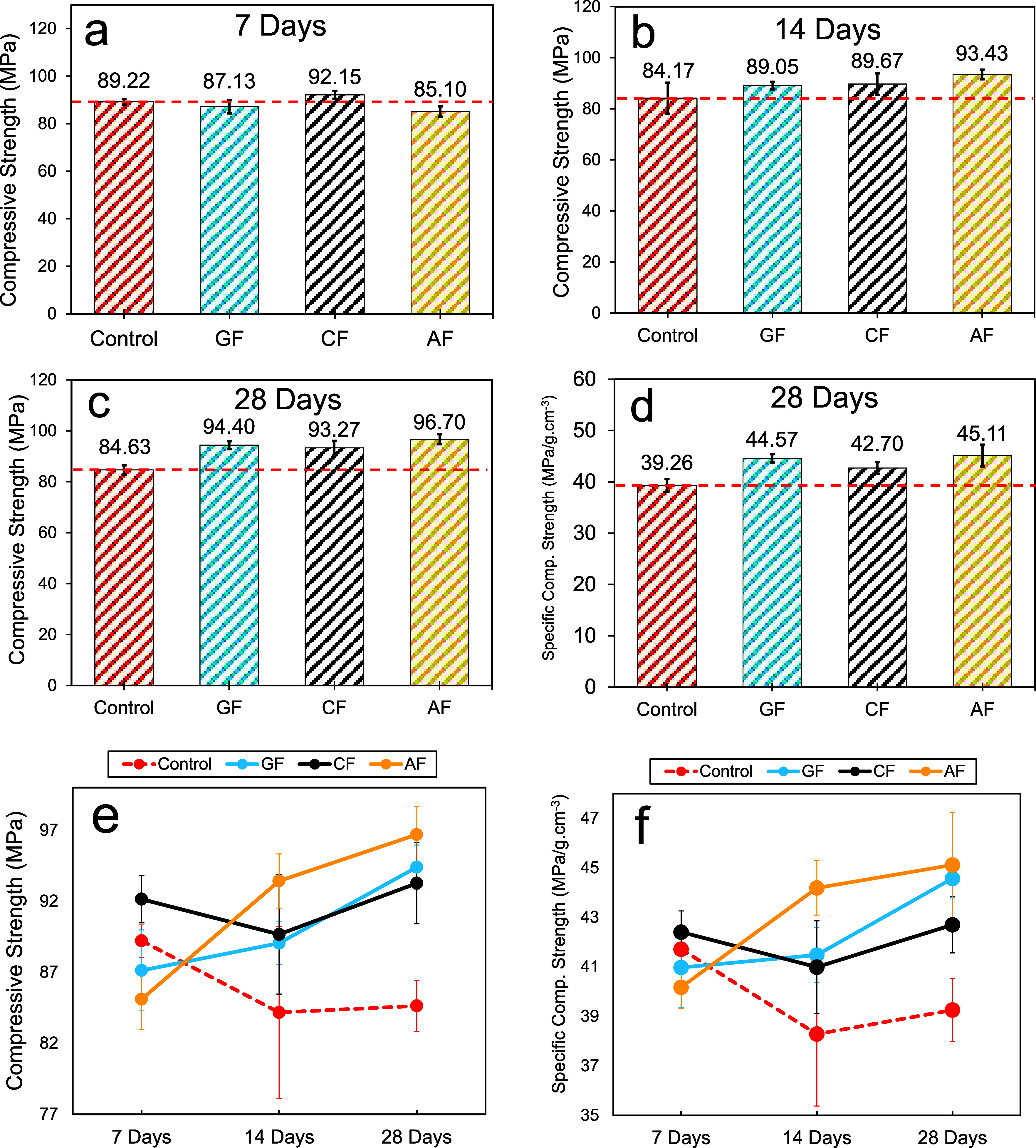
Average compressive strength: (a) 7 days, (b) 14 days, (c) 28 days, (d) specific compressive strength for 28 days, (e) compressive strength variations depending on curing times, and (f) specific compressive strength variations depending on curing times.

After 7 days of curing, no significant improvement in the compressive strength of the fabric-reinforced specimens was observed. The reason is thought to be that there was not yet good adhesion between the fabrics and polymer concrete during this period. Based on these results, it is also understood that the CF fabric shows slightly better compression performance. As mentioned before, this is due to the better adhesion of the CF fabrics to the polymer concrete. It can be said that the compression behavior of the specimens became stable after a curing time of 14 days. During this time, the polymer concrete hardened, and the interface formed with the fabric became more stable. Improvements after 28 days of curing time indicate that the interfacial strength between the cured PC and fabric has peaked. It is assumed that all fabrics positively affect the compressive behavior of the specimens and contribute positively to the compressive strength by increasing the load-bearing capacity of the specimen under compression. Similar studies in the literature have generally focused on the compression behavior of short fiber-reinforced polymer concretes. In a study on chopped glass fiber reinforcement, it was reported that a decreasing trend in the compressive strength of polymer concrete with glass fiber reinforcement was observed.^25^ A study on natural fiber-reinforced polymer concretes reported that hemp and wool fiber reinforcement at different reinforcement ratios caused either a decrease or an increase in the compressive strength of polymer concrete depending on the reinforcement ratio.^19^ On the other hand, another study^2^ found a 16% increase in the compressive strength of chopped carbon fiber-reinforced polymer concretes and an 8.7% increase in the compressive strength of chopped glass fiber-reinforced specimens. In the literature, chopped fiber is generally used for reinforcement of polymer concretes, and it is observed that increasing fiber volume fraction directly affects compressive strength. Matrix/reinforcement dispersion is an important parameter that directly affects the mechanical properties of the composite. Increasing the fiber volume fraction may cause agglomeration and clusters in polymer concrete, adversely affecting the compressive strength. A homogeneous well dispersion may result in more effective compression behavior. Using the woven fabric utilized in this study, a regular and controlled reinforcement process was achieved instead of good dispersion requirement. Figure 12 shows images of the specimens as a result of the compression test. Correspondingly, Figure 12d shows a cross-sectional view of the interface. Figure 12f shows the upper surfaces after compression load. From these images, it is understood that the compressive stresses are not concentrated at the edges of the specimen and are distributed homogeneously over the surface. It was also observed that the PC specimens exhibited brittle behavior in compression.

### 3.7 Specific Strength Results

Specific strengths that evaluate two critical properties, lightweight and strength parameters together, are essential for engineering composites. This phenomenon also plays a critical role in concrete structures and has been thoroughly analyzed in previous studies.^41,42^ Therefore, specific strengths were calculated for both of the mechanical tests. Figures 11d and 13d show the average specific flexural and compressive strength results of the specimens after 28 days of curing. In addition, Figures 11f and 13f show the variation of specific strengths depending on curing times. The specific strengths were calculated by dividing the flexural and compressive strengths of the specimens by their densities. According to these results, at the end of the 28-day curing period, the average specific flexural strengths of control, AF, GF, and CF specimens were 8.96 ± 1.56, 10.20 ± 0.19, 10.12 ± 0.25, and 10.08 ± 0.40 MPa, respectively, while average specific compressive strengths were 39.26 ± 1.28, 45.11 ± 2.11, 44.57 ± 0.79, and 42.70 ± 1.14 MPa, respectively. Consequently, the highest performance in terms of specific flexural strength was observed in the AF specimens, and a 14% increase in the average specific flexural strength of these specimens was recorded compared to that of the control specimen. In other fabric-reinforced composites, an increasing trend was observed at values very close to this rate. The increasing trend in the specific compressive strength of fabric-reinforced composites also continued. In this context, the most significant increase was observed in the AF specimens, with an increase in average specific compressive strength of approximately 15% compared to the control specimen. These results show that the woven fabric reinforcement reduced the density of the polymer concrete, particularly in the AF and GF specimens, and at the same time increased the strength in all specimens, resulting in an increase in the proportion calculation, i.e., an increase in the specific strengths. In other words, woven fabric reinforcement generally lightens the polymer concrete while increasing its strength performance. These results are necessary and important parameters for a lightweight and high-strength structure.

## 4 Conclusion

Fiber reinforcement of polymer concretes to increase the strength of PCs is mainly concentrated on short/chopped fibers. In previous studies, these fibers have shown significant results and improved the mechanical properties of polymer concretes. However, it has been observed that the internal reinforcement of woven fabrics in polymer concretes has not been widely investigated in the literature. This study investigated the mechanical behavior of polymer concretes reinforced with aramid, glass, and carbon woven fabrics. First, the mechanical properties of the fabrics used in the reinforcement processes were investigated, and then, the density, flexural, and compression tests of the reinforced polymer concrete specimens were carried out. The following results were obtained.

Aramid fabric has mechanical properties superior to those of the fabrics used in the study. The yarns in the aramid fabrics exhibited better pull-out behavior than the other fabrics due to their high frictional performance. In addition, the high single yarn tensile load of aramid fabrics compared to other fabrics, combined with the high yarn pull-out load, resulted in the woven tensile load being superior to those of other fabrics. Carbon fabric is the second most efficient among the fabrics used in the study. Carbon fabric has attracted attention with its high single yarn and woven tensile performance. However, it should be noted that the pull-out load of glass fabric is higher than that of carbon fabric. Within the scope of these results, the single yarn tensile load of aramid fabric is 216% and 21%, the yarn pull-out loads are 40% and 53%, and woven fabric tensile loads are 60% and 20% higher than that of glass and carbon fabrics, respectively.

The highest average density was observed in the CF specimens as a result of the 28 days of curing time with carbon woven fabric reinforcement. It is believed that the CF reinforcement forms a better interface with the polymer concrete, and the voids at the interfaces of these specimens are less than those of the specimens reinforced with other fabrics. The lowest average density was observed in the GF specimens, and it was understood that the average densities of these specimens were 1.82%, 3.04%, and 1.30% lower than those of the control, CF, and AF specimens, respectively. This slight decrease in density with a single layer of woven fabric reinforcement is remarkable. It is also thought that even lower density values can be achieved by increasing the number of reinforced layers in the GF and AF samples.

Woven fabric reinforcement improved the flexural and compressive behaviors of polymer concrete. As a result of the 28-day curing period, the average flexural strength of the CF-reinforced specimen increased by 14% compared with the control specimen, while the average compressive strength of the AF-reinforced specimen increased by 14%. With a 28 day curing period, increases of at least 10% were achieved for all fabric types in both flexural and compression tests. Fabrics carried the load in polymer concretes under bending and increased the flexural strength of the specimens by creating a bridging effect. Similarly, the fabrics contributed to the compressive load and increased the compressive strength of the specimens. On the other hand, the average specific flexural and compressive strengths of the reinforced specimens increased compared to the control specimens after 28 days of curing. In this context, the specific flexural and compressive strengths of the AF specimens increased by 14% and 15%, respectively, compared to those of the control specimen. The glass woven fabric reinforcement decreased the density of the polymer concrete and improved the performance of the specific strength of the GF specimens.

Consequently, with its superior mechanical properties, the aramid fabric showed better performance than the other fabrics for polymer concrete. However, the glass woven fabric reinforcement reduced the density of the polymer concrete and increased the specific strength of the specimen. Carbon fabric improved the flexural strength by providing a good interface with polymer concrete. The study results showed that dry-woven fabrics could improve the mechanical properties of polymer concrete and that this process can be carried out quite manageably and effortlessly. Polymer concretes reinforced with woven fabrics, manufactured with an effective and practical strengthening method, may be incorporated into sustainable systems by using them in lightweight prefabricated and smart structures, restoration and strengthening of historical structures, and infrastructure elements. In addition, the effect of multilayer woven fabric reinforcement on the mechanical properties of polymer concretes needs to be investigated in further studies on the topic.
